# Nanobody‑horseradish peroxidase and -EGFP fusions as reagents to detect porcine parvovirus in the immunoassays

**DOI:** 10.1186/s12951-019-0568-x

**Published:** 2020-01-07

**Authors:** Qizhong Lu, Xiaoxuan Li, Jiakai Zhao, Jiahong Zhu, Yuhang Luo, Hong Duan, Pinpin Ji, Kun Wang, Baoyuan Liu, Xueting Wang, Wenqi Fan, Yani Sun, En-Min Zhou, Qin Zhao

**Affiliations:** 1 Department of Preventive Veterinary Medicine, College of Veterinary Medicine, Northwest A&F University, Yangling, 712100 Shaanxi China; 2 Scientific Observing and Experimental Station of Veterinary Pharmacology and Diagnostic Technology, Ministry of Agriculture, Yangling, 712100 Shaanxi China

**Keywords:** Nanobody, Nanobody-HRP, Nanobody-EGFP, Porcine parvovirus, VP2

## Abstract

**Background:**

Antibodies are an important reagent to determine the specificity and accuracy of diagnostic immunoassays for various diseases. However, traditional antibodies have several shortcomings due to their limited abundance, difficulty in permanent storage, and required use of a secondary antibody. Nanobodies, which are derived from single-chain camelid antibodies, can circumvent many of these limitations and, thus, appear to be a promising substitute. In the presented study, a sandwich ELISA-like immunoassay and direct fluorescent assay with high sensitivity, good specificity, and easy operation were the first time to develop for detecting porcine parvovirus (PPV). After screening PPV viral particles 2 (VP2) specific nanobodies, horseradish peroxidase (HRP) and enhanced green fluorescent protein (EGFP) fusions were derived from the nanobodies by recombinant technology. Finally, using the nanobody-HRP and -EGFP fusions as probes, the developed immunoassays demonstrate specific, sensitive, and rapid detection of PPV.

**Results:**

In the study, five PPV-VP2 specific nanobodies screened from an immunised Bactrian camel were successfully expressed with the bacterial system and purified with a Ni–NTA column. Based on the reporter-nanobody platform, HRP and EGFP fusions were separately produced by transfection of HEK293T cells. A sandwich ELISA-like assay for detecting PPV in the samples was firstly developed using PPV-VP2-Nb19 as the capture antibody and PPV-VP2-Nb56-HRP fusions as the detection antibody. The assay showed 92.1% agreement with real-time PCR and can be universally used to surveil PPV infection in the pig flock. In addition, a direct fluorescent assay using PPV-VP2-Nb12-EGFP fusion as a probe was developed to detect PPV in ST cells. The assay showed 81.5% agreement with real-time PCR and can be used in laboratory tests.

**Conclusions:**

For the first time, five PPV-VP2 specific nanobody-HRP and -EGFP fusions were produced as reagents for developing immunoassays. A sandwich ELISA-like immunoassay using PPV-VP2-Nb19 as the capture antibody and PPV-VP2-Nb56-HRP fusion as the detection antibody was the first time to develop for detecting PPV in different samples. Results showed that the immunoassay can be universally used to surveil PPV infection in pig flock. A direct fluorescent assay using PPV-VP2-Nb12-EGFP as a probe was also developed to detect PPV in ST cells. The two developed immunoassays eliminate the use of commercial secondary antibodies and shorten detection time. Meanwhile, both assays display great developmental prospect for further commercial production and application.

## Background

For diagnostic and detection purposes, antibody-mediated immunoassays offer a specific and accurate detection method for antigens and are universally used in laboratories and clinical diagnosis. To date, numerous antibodies against different antigens have been produced for clinical application; specifically, traditional polyclonal and monoclonal antibodies are the most commonly used [1–5]. Nevertheless, traditional antibodies have their limitations as reagents for developing diagnostic immunoassays, including the required affinity purification of monospecific antibodies from sera, labels, such as horseradish peroxidase (HRP) and fluorescence, and the use of secondary antibodies. More recently, single-chain antibodies derived from camelids, named nanobodies, possess antigen-recognition sites that can be easily expressed with different systems, thus offering an effective detection method for diagnostic purposes [6–8]. Because nanobodies contain only one ∼ 130 amino acid variable domain, they can be simply derivatised by coupling to reporters or dyes. For example, one study designed a reporter-nanobody fusion (RANbody) platform, in which RANbody was used in immunohistochemical detection [9]. Other works have reported the application of nanobody-HRP, EGFP, or nano-luciferase fusions derived from nanobodies to develop detection assays, label cells and tissues, and for other purposes [10–13].

Porcine parvovirus (PPV) is a major pathogen causing reproductive failure in sows, which is revealed by early embryonic death, fetal cadaveric death, stillbirth, infertility, and delayed estrus [14–16]. In addition, some reports suggested that PPV can cause diarrhea and dermatitis in piglets, and co-infection with porcine circovirus type 2 (PCV2) can enhance the multi-systemic wasting syndrome in weaned piglets [15]. Thus, PPV infection has caused detrimental consequences in the pig industry, such as economic decline. Although the virus has been classified into four clinical genotypes, there is currently only one serotype of PPV [17]. PPV is a non-encapsulated autonomously replicating virus that belongs to the family *Parvovirdae*, subfamily *Parvovirina*, and genus *Parvovirus* [18]. The same genus also includes parvoviruses of cattle, cats, dogs, geese, mice, rats, tigers, rabbits, minks, chickens and raccoons [19–24]. The PPV genome is a single and negative-stranded DNA with a full length of about 5000 bp, which contains two open reading frames (ORFs) and covers the entire genome [23, 25]. Out of which, ORF2 encodes viral structural proteins, including viral particles 1 (VP1), VP2, and VP3 with molecular weights of 83, 64, and 60 kDa, respectively [26, 27]. VP2 is the main structural and immunogenic protein of PPV that possesses neutralising antigenic epitopes and hemagglutination sites of viruses. These features promote VP2 as a primary target for designing the serology diagnosis assay and subunit vaccines [28–30].

**Scheme 1:**
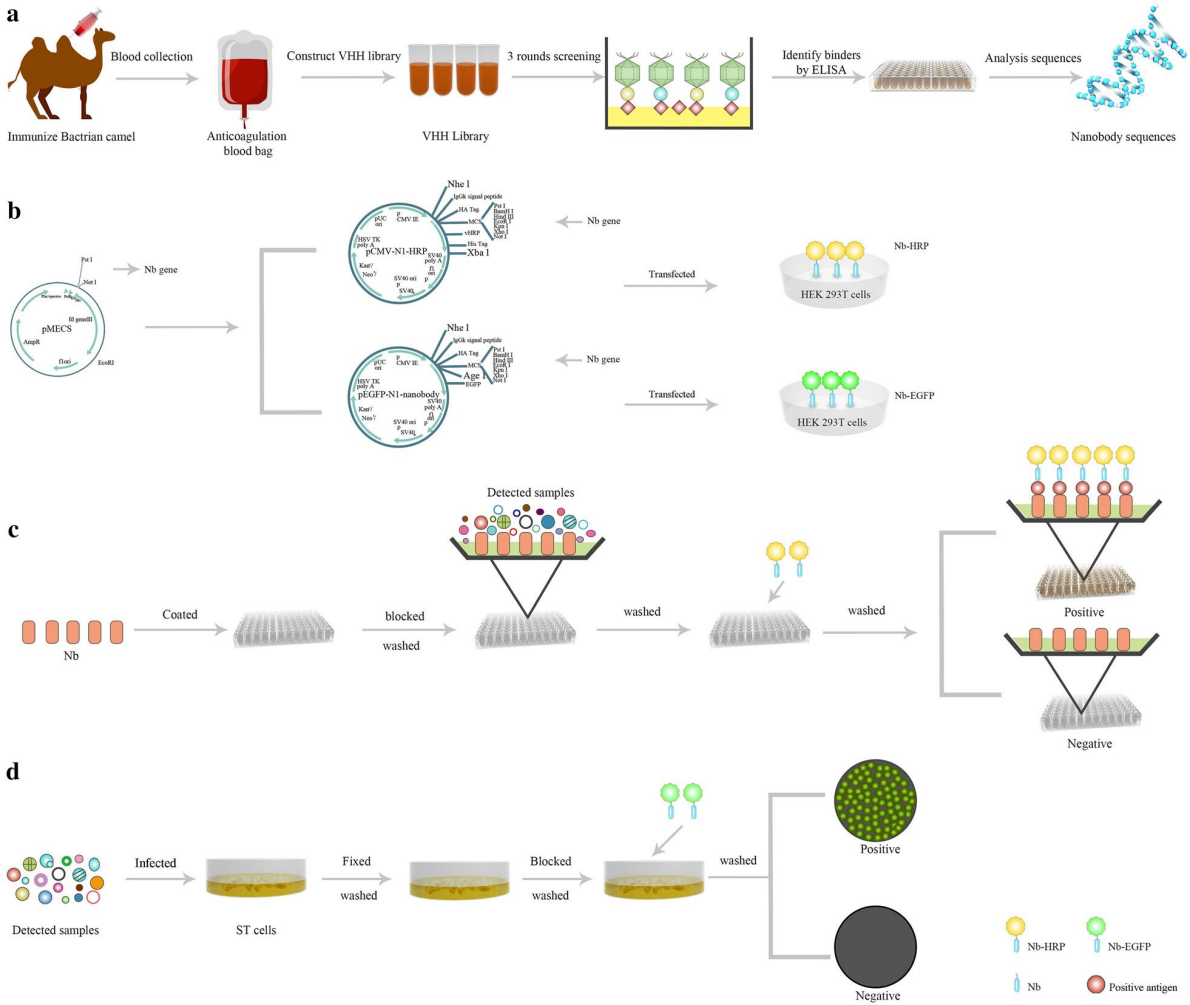
Schematic representation of screening the nanobodies and detection porcine parvovirus with reporter-nanobody fusions. **a** Screening the nanobodies from an immunized phage display library. **b** The platform for expressing nanobody-HRP and nanobody-EGFP fusion proteins. **c** Detection porcine parvovirus with nanobody-HRP fusions. **d** Detection porcine parvovirus with nanobody-EGFP fusions

The currently available assays for detecting PPV include virus isolation, indirect fluorescent assay (IFA), haemagglutination test, enzyme-linked immunosorbent assay (ELISA), polymerase chain reaction (PCR), real-time PCR, and others [14, 31–39]. Among these, PCR and real-time PCR are the most universally employed because of their high sensitivity [34, 35]. Yet, the two assays require complicated operation and easily produce false-positive results due to cross-contamination [33]. While traditional antibody-based ELISAs are also widely used to detect the antibodies against PPV and viral particles, the need for a secondary antibody and enzyme labels results in a complicated manufacturing process and high cost [32, 37, 38]. In the present study, to develop an enhanced immunoassay for detecting PPV, PPV-VP2 specific nanobodies were screened and produced from an immunised Bactrian camel by phage display technology (Scheme 1a). Based on the production platform of the reporter-nanobody, PPV-VP2 specific nanobody-HRP and -EGFP fusions were then expressed (Scheme 1b). When designed the sandwich ELISA-like immunoassay to detect PPV, the nanobody was utilised as the capture antibody and nanobody-HRP fusion as the detection antibody (Scheme 1c). To develop the direct fluorescent assay for detecting PPV in cells, nanobody-EGFP was used as a probe (Scheme 1d). Both assays exhibited high agreement with real-time PCR and could effectively detect PPV in clinical samples. Importantly, the two assays do not require use of a secondary antibody, enzymes, or fluorescence labels and can be easily produced for future commercial application.

## Materials and methods

### Cells, virus and vectors

ST and HEK293T cell lines were purchased from ATCC and cultured in Dulbecco’s Modified Eagle’s Medium (Life Technologies Corp, USA) containing 10% fetal bovine serum (FBS, Gibco, USA) at 37 °C in 5% CO_2_. ST cells were used to propagate PPV (strain 7909) as previously described [28], and HEK293T cells were used to express the recombinant nanobodies fused with HRP and EGFP. The PPV stocks were proliferated in the ST cells and had the 10^−5.5^/mL of TCID_50_. The pET-28a and pET-25b vectors (Novagen, USA) were separately used for prokaryotic expression of the PPV-VP2 protein and nanobodies. The pMECS vector was used to construct phage display library. The pCMV-N1-HRP vector described in a previous study was utilised to produce nanobody-HRP fusions [11]. The pEGFP-N1 vector (Clontech, Japan) was employed as a backbone to construct the platform for nanobody-EGFP fusions.

### Expression, purification and identification of PPV-VP2 recombinant protein

To express the recombinant PPV-VP2 protein, the complete VP2 gene from the PPV (7909) strain (GenBank accession number AY583318) was synthesised (GENEWIZ Company, Jiangsu, China). To express the soluble PPV-VP2 protein, the target and chaperone Tf16 proteins were co-expressed in the *E. coli* strain BL21 (DE3) based on a previous description [28]. Briefly, the recombinant plasmid was constructed. Then, the VP2 gene was amplified by PCR with primer pairs (28a-VP2-Forward: 5′-CCC**GGATCC**ATGAGTGAAAATGTGGAA-3′; 28a-VP2-Reverse: 5′-CCC**CTCGAG**GTATAATTTTCTTGGTAT-3′, bold sequences were separately *Bam*H I and *Xho* I) using the synthesised VP2 gene as the template. Subsequently, the PCR products and blank pET-28a vector (Novagen, USA) were digested with the same enzymes *Bam*H I and *Xho* I, and the two digested products were ligated to construct the recombinant pET-28a-VP2 plasmid. The positive plasmids were sequenced and analysed by MegAlign software to determine the successful construction. Secondly, the commercial chaperone plasmid Tf16 (TaKaRa, China) was transformed into *E. coli* strain BL21 (DE3), then the competent cells BL21 (DE3)-Tf16 were prepared according to the manufacturer’s protocol (TaKaRa, China). Next, the positive plasmid pET-28a-VP2 was transformed into the BL21 (DE3)-Tf16 competent cells. Finally, the positive recombinant *E. coli* was induced by the addition of 0.1 mM isopropyl-β-d-thiogalactoside (IPTG) and 2 mg/mL L-arabinose for co-expression of PPV-VP2 and Tf16 proteins. After the bacteria were sonicated, the supernatant containing PPV-VP2 protein was purified by Ni–NTA Beads 6FF Agarose (SMART, Changzhou, China). SDS-PAGE and Western blot assays were used to analyse the expression, purity, and antigenicity of the recombinant PPV-VP2 protein.

### Immunisation of Bactrian camel and construction of VHH library

A healthy 4-year-old Bactrian camel was immunised with the purified recombinant PPV-VP2 protein based on previously reported procedures [40, 41]. Briefly, 2 mg recombinant PPV-VP2 protein (1 mg/mL) was mixed with an equal volume of Freund’s complete adjuvant for the first immunisation and with the same volume of Freund’s incomplete adjuvant for the following four immunisations. The titration of the antibody against the PPV-VP2 protein in the serum samples from the last immunisation was detected with an indirect ELISA using the recombinant PPV-VP2 protein as the coating antigen.

After the last immunisation, the peripheral blood lymphocytes (PBLs) were extracted from 250 mL blood sample by Leucosep^®^ tubes (Greiner Bio-One, Germany) for library construction. Total mRNA was extracted from 5 × 10^7^ PBLs and used for cDNA synthesis by reverse transcriptase with the Olig (dT)_18_ primer. The VHH genes were amplified with the nested PCR using primer pairs CALL001 (5′-GTCCTGGCTGCTCTTCTACAAGG-3′), CALL002 (5′-GGTACGTGCTGTTGAACTGTTCC-3′) and VHH-FOR (5′-CAGGTGCAGCTGCAGGAGTCTGGGGGAGR-3′), VHH-REV (5′-CTAGTGCGGCCGCTGAGGAGACGGTGACCTGGGT-3′) in order to avoid contamination by the VH genes, according to a previous description [40]. Then, the nested PCR products were ligated into phagemid vector pMECS through the same PstI and NotI (underline in the primers) endonucleases digestion. The recombinant phagemids were electro-transformed into freshly competent *E. coli* TG1 cells, and the positive rate of the constructed library was determined by PCR amplification with primers MP57 (5′-TTATGCTTCCGGCTCGTATG-3′) and VHH-REV [41]. Finally, 48 clones were randomly selected for sequencing to analyse the library’s diversity.

### Screening and identification specific nanobodies against PPV-VP2 protein

To select nanobodies against the PPV-VP2 protein, three rounds of screening and phage rescuing were performed with an indirect ELISA, according to a previous description [40]. For bio-panning, the purified PPV-VP2 protein was used as the coating antigen in the indirect ELISA. After three rounds of screening, the PPV-VP2 specific phage particles were enriched and evaluated with polyclonal phage ELISA. Then, 96 randomly selected clones were grown in liquid culture, and their periplasmic extracts were tested by the indirect ELISA for detecting the presence of specific nanobodies against the PP2-VP2 protein. Finally, all positive clones were sequenced and classified based on their complementary determining regions (CDRs) amino acid sequence.

### Expression and purification of different nanobodies against PPV-VP2 protein

To express the above screened nanobodies, the pET-25b vector (Novagen, USA) was used. Firstly, the VHH genes encoding nanobodies were amplified with the pMECS plasmid from *E. coli* TG1 cells as a template using the following primers: (VP2-Nbs-F: 5′-TATGGATCCGCAGGTGCAGCTGCAGGAG-3′; VP2-Nbs-R: 5′-AGTAAGCTTTGAGGAGACGGTGACCTG-3′). Then, the PCR products were digested with enzymes BamHI and HindIII (underline in the primers) and ligated into pET-25b vectors digested with the same two enzymes. The positive plasmids were also sequenced and analysed by MegAlign software to confirm the successful construction. Subsequently, the recombinant positive plasmids were transformed into *E. coli* BL21 (DE3) competent cells for expression by induction of 0.1 mM IPTG. The expressed nanobodies were purified by immobilised metal affinity chromatography (IMAC) using Ni–NTA (SMART, Changzhou, China) based on the instructions of the manuscript. SDS-PAGE was used to analyse the expression and purification of nanobodies.

### Expression of nanobody-HRP and -EGFP fusions against PPV-VP2 protein

The nanobody-HRP fusions were expressed in HEK293T cells based on a previous study [11]. Briefly, the VHH genes were obtained from the positive pMECS plasmids through the digestion of PstI sites and *Not* I enzymes and ligated into the modified pCMV-N1-HRP vector digested with the same two enzymes. Then, the positive plasmids were transfected into the HEK293T cells with polyetherimide agents (PEI, Polysciences Inc. Warrington, USA). The cell culture medium containing secreted nanobody-HRP fusions was harvested and filtered through 0.45-μm cellulose acetate membranes for direct use. Both indirect immunofluorescent assay (IFA) and direct ELISA were used to determine the expressions of nanobody-HRP fusions in the HEK293T cells and the fusions secreted into the medium. Direct ELISA, employing the medium as the coating antigen, indicated the presence of the fusion via a colour change and was used to detect the titres of nanobody-HRP fusions against the PPV-VP2 protein in the medium.

To produce the nanobody-EGFP fusions, the expression platforms were designed based on the construction of the pCMV-N1-HRP vector [11]. Briefly, the primer pairs (EGFP-nanobody-F: 5′-CTGGCTAGCATGGAGACCGACA-3′, EGFP-nanobody-R: 5′-TGAACCGGTGGACCACTGCCACTACTACTGGC-3′) were designed to amplify a secreting signal sequence (the human IgG kappa chain), HA tag, multiple cloning site (MCS), and a short linker from the modified pCMV-N1-HRP vector. Then, the PCR products were digested with two enzymes, NheI and AgeI, and ligated into the commercial vector pEGFP-N1 digested with the same enzymes. The modified vector was named pEGFP-N1-nanobody. Next, the VHH genes were also obtained from the pMECS plasmids and cloned into the pEGFP-N1-nanobody vector. The positive plasmids were transfected into HEK293T cells, and the expressions of nanobody-EGFP fusions were detected by direct observation via fluorescence microscopy (Leica AF6000, Germany). Western blot assay was used to confirm that the nanobody-EGFP fusions were secreted into the medium. The medium was concentrated and used to directly run SDS-PAGE. The anti-HA monoclonal antibody, as the first antibody, and HRP-labelled goat-mouse antibody, as the second antibody, were used for Western blot analysis. The titres of nanobody-EGFP fusions against the PPV-VP2 protein in the medium were detected with indirect ELISA.

In addition, IFA and FA were separately employed to determine whether the nanobody-HRP and -EGFP fusions could be used to detect PPV infection in ST cells.

### Indirect ELISA

The indirect ELISA was used to detect the titres in serum samples from the immunised camel, screen nanobodies against PPV-VP2 protein, and determine the titres of prokaryotic expressed nanobodies and nanobody-EGFP fusion proteins. The ELISA plates were coated with the purified PPV-VP2 protein and incubated overnight at 4 °C. After washed three times with PBS’T (0.5% Tween-20 in PBS), the plates were blocked with the blocking buffer (2.5% skimmed-milk in PBS’T). For the titres of serum samples, sera of different dilutions were added to the plates followed by the addition of the rabbit anti-camel antibody and HRP-conjugated goat anti-rabbit antibody. To screen nanobodies and determine the titres of prokaryotic expression of nanobodies, the anti-HSV monoclonal antibody and HRP-conjugated goat anti-mouse IgG were added to the plates after the supplementation of nanobodies. For nanobody-HRP fusions, the reactions were indicated by a colour change. For nanobody-EGFP fusions, the anti-HA monoclonal antibody and HRP-conjugated goat anti-mouse IgG were added. The reaction was coloured with the tetramethylbenzidine (TMB) [A: 205 mM Potassium Cirate (pH 4.0); B: 41 mM tetramethyl benzidine; A:B (v/v) = 39:1]. After the reaction was stopped with 3 M H_2_SO_4_, the optical density at 450 nm (OD_450nm_) was read using an automatic ELISA plate reader.

### Immunofluorescent assay

To identify the nanobody-HRP fusions expressed in HEK293T cells, the transfected cells were fixed with 70% ice-ethanol followed by supplementation of the anti-His monoclonal antibody and FITC-conjugated goat anti-mouse antibody. To verify the binding of nanobody-HRP and -EGFP fusions to PPV, the ST cells were inoculated with 100 TCID_50_ PPV (TCID_50_ = 10^−4.5^/100 μL) when 40–60% confluence was reached in a 96-well plate. After incubation for 2 h at 37 °C in 5% CO_2_, the cells were washed three times with PBS. After 48 h of infection, the infected cells were fixed with 70% ice-ethanol for 30 min at room temperature (RT). Then, the cells were blocked with 1% bovine serum albumin (BSA, BioFoX, Germany) and subsequently washed with PBS. After the nanobody-HRP fusions were incubated with the ST cells for 1 h at 37 °C, the anti-His monoclonal antibody and Cy3-conjugated goat anti-mouse antibody were added. Finally, the stained cells were analysed by fluorescence microscopy (Leica AF6000, Germany). The positive and negative pig sera against PPV were used as the respective positive and negative controls and the FITC-conjugated goat anti-swine antibody as the secondary antibody. After the nanobody-EGFP fusions were incubated for 1 h at 37 °C, the cells were directly analysed by fluorescence microscopy.

### Nanobody-EGFP fusion as a probe to detect the different PPV isolates by direct immunofluorescent assay

To determine whether the nanobody-EGFP fusions can be used to detect different PPV isolates by direct immunofluorescent assay, 12 clinical PPV isolates were inoculated into the ST cells. After 48 h of infection, the infected cells were fixed with 70% ice-ethanol then the selected nanobody-EGFP fusions were added. After incubation for 1 h at RT, the cells were directly observed under fluorescence microscopy.

### Nanobody-HRP fusion as a probe for developing the sandwich ELISA-like immunoassay

To analyse the nanobody-HRP fusions as a probe to detect PPV, a sandwich ELISA-like assay was developed using nanobody as the capture antibody and nanobody-HRP fusions as the detection antibody. First, the best pairs of nanobodies and nanobody-fusions were determined by the orthogonal assay. The same amount (1000 ng/well) of different nanobodies was coated in the ELISA plate. After the plates were blocked with 1% gelatin, one TCID_50_ PPV stock (Positive, P) was added into each well and incubated overnight at 4 °C. The medium from normal ST cells was used as the negative control (N). After washed three times with PBS’T, the same dilutions of different nanobody-HRP fusion proteins were added to the wells and incubated for 1 h at 37 °C. After washed three times again, the plates were supplemented with TMB substrate, and the OD_450nm_ value was read after the reaction was stopped with 3 M H_2_SO_4_. The best pairs were selected when the highest numerical values of P/N were obtained.

The optimal amount of capture nanobody and dilution of detection nanobody-HRP fusions for the sandwich ELISA-like assay were determined using a checkerboard titration. Different amounts of the capture nanobody, including 1000, 2000, 4000 and 8000 ng/well, and dilution ratios of 1:1, 1:10, 1:100, 1:1000, and 1:10,000 for the detection nanobody-HRP fusion proteins were used in the assay. One TCID_50_ PPV viral stock was employed as the positive and the same volume of medium from normal ST cells as the negative. Then, the optimal amount of capture nanobody and detection nanobody-HRP fusion proteins were determined when the numerical values of P/N were the highest.

### Validation of the developed sandwich ELISA-like immunoassay

To determine the cut-off value of the sandwich ELISA-like assay, 200 negative samples, including 40 medium samples from different passage ST cells, 80 serum samples, and 80 fecal samples were detected. The cut-off value was set at the mean OD_450nm_ values of the 200 negative samples plus 3 standard deviations (SD) to ensure 99% confidence for the negative sera samples within this range.

To determine the specificity of the assay, various swine viruses, including porcine reproductive and respiratory syndrome (PRRSV), porcine epidemic diarrhea virus (PEDV), porcine circovirus type 2 (PCV2), porcine pseudorabies virus (PRV) and transmissible gastroenteritis virus (TGEV), were used for testing.

To determine the low limitation of the assay for detecting PPV particles and VP2 proteins, 10 dilutions of PPV stocks and VP2 proteins were detected. In addition, the gene copies of lowest PPV particles were also determined by real-time PCR as described below.

To evaluate the developed assay for detecting PPV in the clinical samples, 64 faeces (n = 43) and sera (n = 21) samples were collected from the diseased pigs for testing.

### Comparisons between the nanobody-HRP and -EGFP fusions for detecting PPV with other developed methods

The above 64 clinical samples were also detected using a commercial monoclonal antibody-based sandwich ELISA kit, real-time PCR, and direct immunofluorescent assay with the nanobody-EGFP fusions developed in the study. For the commercial monoclonal antibody-based sandwich ELISA kit, the procedures were performed according to the operation instructions. For real-time PCR, the following primer and probe were designed using the Primer 5.0 software based on the VP2 gene sequences of the PPV strain (GenBank accession number AY583318): VP2-F: 5′-CAAGCAATATTCAATGTAGTAC-3′, VP2-R: 5′-GCTTGCAGTTAGATCATTA-3′) and the TaqMan probe 5′-(FAM) AGAATCAGCAACCTCACCACCA (TAMRA)-3′. The Premix Ex Taq (Probe qPCR) (TaKaRa, Dalian, China) were used to construct the reaction mixture of real-time PCR assay based on the instructions. The reaction condition was 95 °C for 30 s, 40 cycles consisting of denaturation at 95 °C for 5 s, annealing at 55 °C for 10 s and extension at 72 °C for 20 s. PCR amplification was performed using the StepOnePlus Real-time PCR System (Applied Biosystems, Thermo Fisher Scientific, USA). All samples were run in duplicate and inoculated with the ST cells for detection of PPV using the direct immunofluorescent assay.

In addition, a total of 42 samples, including 21 faeces and 21 sera, were prepared from the 3 pigs infected with PPV stock by oral routes at 3, 5, 7, 10, 14, 21, and 28 days post inoculation (dpi). Then, detection of PPV was performed with the developed sandwich ELISA-like assay using nanobody-HRP fusion as a probe, direct immunofluorescent assay with nanobody-EGFP as a probe, real-time PCR, and commercial monoclonal antibody-based sandwich ELISA for comparison.

### Statistical analysis

The statistical differences were evaluated by Student’s *t* test for the two groups. Data presentation was performed using GraphPad Prism version 5.0 (GraphPad Software, San Diego, CA, USA). All presented data were shown as the mean ± SD, where *p < 0.05; **p < 0.01; ***p < 0.001; and NS means no significant difference. The Kappa values were calculated to estimate the coincidence between the developed sandwich ELISA-like assay, direct immunofluorescent assay, real-time PCR, and commercial monoclonal antibody-based ELISA kit. These calculations were performed using SPSS software (Version 20, http://www.spss.com.cn).

## Results

### Preparation of the PPV-VP2 recombinant protein

**Fig. 1:**
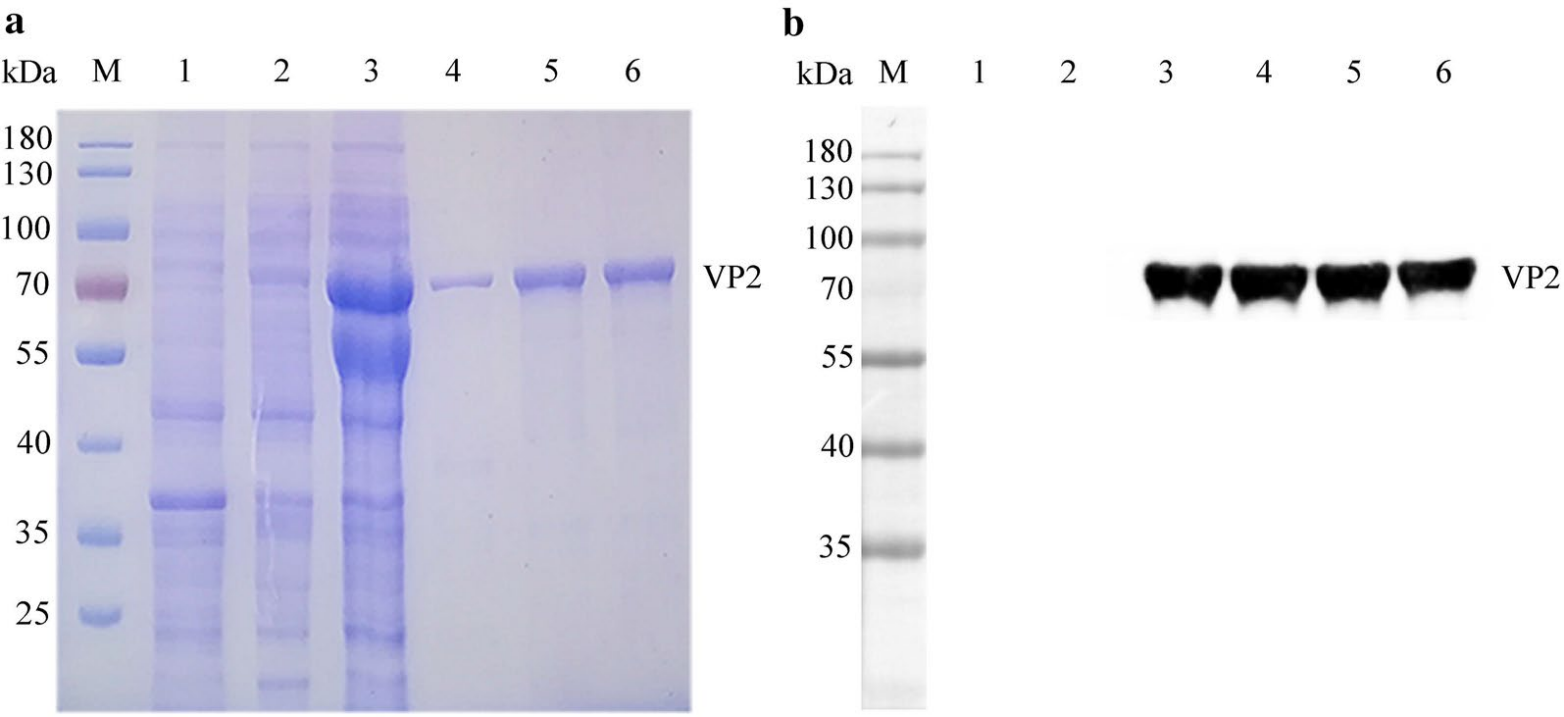
Expression, purification, and identification of the recombinant PPV-VP2 protein. **a** SDS-PAGE analysis of the protein expression and purification. **b** Western blot analysis of the antigenicity of the protein. M: Marker; lane 1: pET-28a vector control; lane 2: un-induced of pET-28a-VP2; lane 3: supernatant after sonication; lane 4: inclusion body; lane 5–6: purified protein

By sequences analysis with MegAlign software, the results showed that the VP2 gene was successfully ligated into the pET-28a vector (Additional file 1: Fig. S1). Using the *E. coli* BL21 (DE3)-Tf16 cells to express, SDS-PAGE analysis showed that the recombinant PPV-VP2 protein was expressed with the expected size of 70 kDa and the highly purified target was obtained after purification (Fig. 1a). Western blot result revealed that the protein could react with the positive swine sera for PPV antibodies, indicating that the expressed and purified PPV-VP2 protein had antigenicity (Fig. 1b).

### Construction of the VHH library

**Fig. 2:**
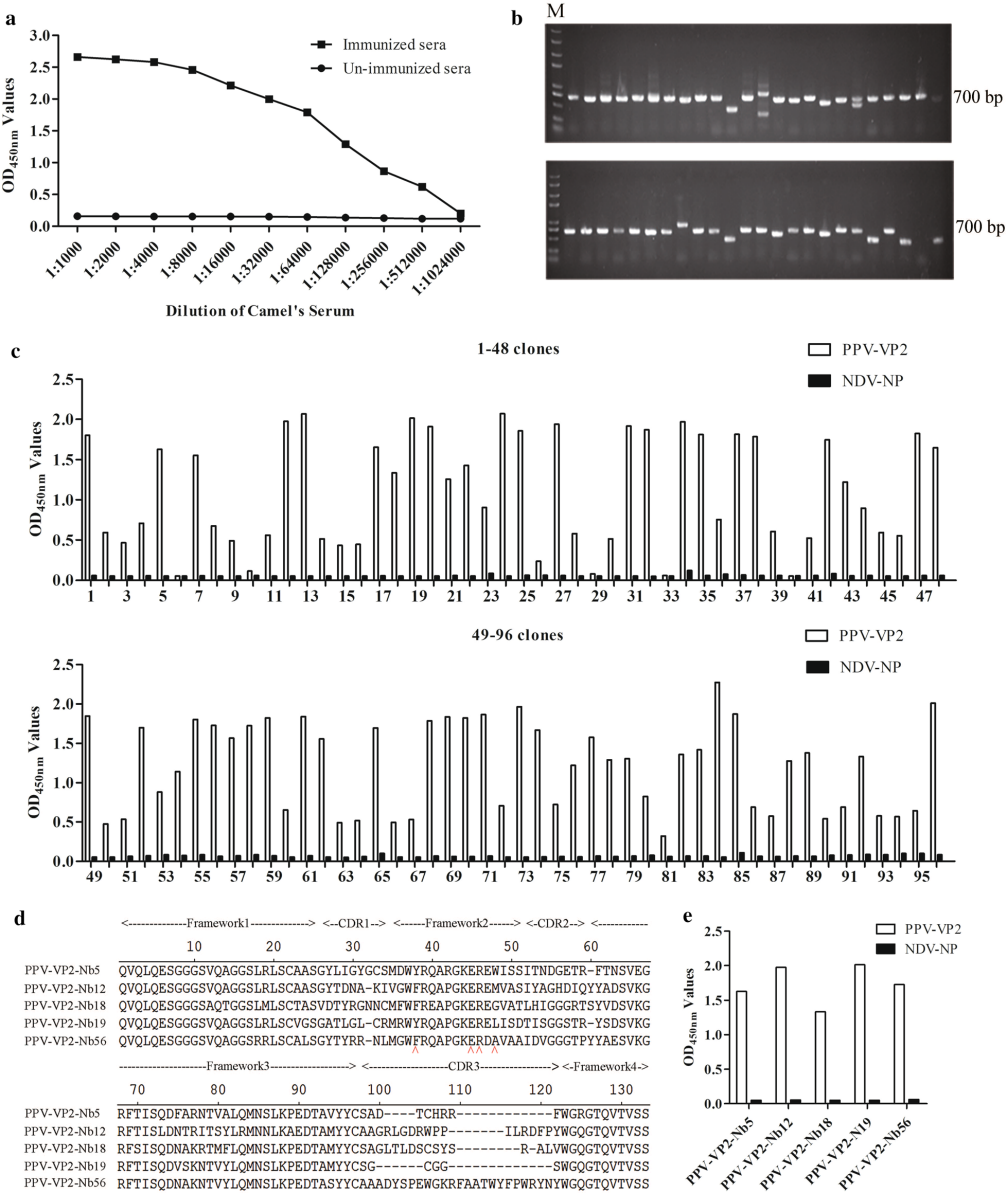
Construction of the VHH library and screening the PPV-VP2 specific nanobodies by phage display technology. **a** Titres of antibodies against PPV-VP2 protein in the sera from the Camel after the fifth immunisation. **b** 48 clones were randomly picked to estimate the correct insertion rate by PCR. The size of PCR products was approximately 700 bp. **c** Identification of the periplasmic extracts from the 96 clones specifically binding to the PPV-VP2 protein with indirect ELISA. 90 clones were identified as positive. **d** Alignment of the amino acid sequences of 5 screened nanobodies against PPV-VP2 protein. The sequences are grouped according to their CDRs. **e** Specific reactions between the 5 screened nanobodies and PPV-VP2 protein

The titres of antibody against PPV-VP2 protein in the serum samples from the immunized camel reached 1:512,000 (Fig. 2a), suggesting that the camel produced a good immune response to the PPV-VP2 protein. After amplification, ligation, and transformation, a phage display VHH library consisting of approximately 3.15 × 10^9^ individual clones was successfully constructed. Then, 48 clones were randomly picked for checking the insertion rate of VHH genes by PCR, which was determined to be 96% (Fig. 2b). Subsequently, the sequences of 48 clones revealed that the library had good diversity (data not shown).

### Screening and identification of specific nanobodies against PPV-VP2 protein

After three rounds of screening, the phages expressing VP2-specific VHHs were enriched and the ratio of positive/negative clones (P/N) increased from 40 to 6.7 × 10^3^ (Table 1). Then, the periplasmic extracts from the 96 randomly selected clones were produced and screened for the specific binding with PPV-VP2 protein. The results revealed that 90 clones could specifically bind with PPV-VP2 protein (Fig. 2c) and were sequenced. According to the amino acid sequences of the CDRs from the 90 clones, the 5 specific nanobodies against PPV-VP2 protein were produced and named PPV-VP2-Nb5, -Nb12, -Nb18, -Nb19, and -Nb56 (Fig. 2d). In addition, sequence alignment indicated that the conserved residues at 37, 44, 45, and 47 positions were all hydrophilic amino acids (Fig. 2d). The indirect ELISA results showed that 5 nanobodies specifically reacted with the PPV-VP2 protein but not with the NDV-NP protein (Fig. 2e). The NDV-NP protein, which was expressed and purified using a similar method to the PPV-VP2 protein, has a 6 × His-Tag, eliminating the possibility that the 5 nanobodies may recognise the 6× His region.

**Table 1:** Enrichment of phage particles against PPV-VP2 specific nanobodies during three rounds of panning

Round of screening	Input (Pfu/well)	P output (Pfu/well)	N output (Pfu/well)	Recovery (P/input)	P/N
1st round	5 × 10^10^	4 × 10^4^	1 × 10^3^	8 × 10^−7^	40
2nd round	5 × 10^10^	8 × 10^6^	5 × 10^3^	1.6 × 10^−4^	1.6 × 10^3^
3rd round	5 × 10^10^	1.95 × 10^9^	2.9 × 10^5^	3.9 × 10^−2^	6.7 × 10^3^

### Expression of the nanobodies against PPV-VP2 protein by the *E. coli* system

**Fig. 3:**
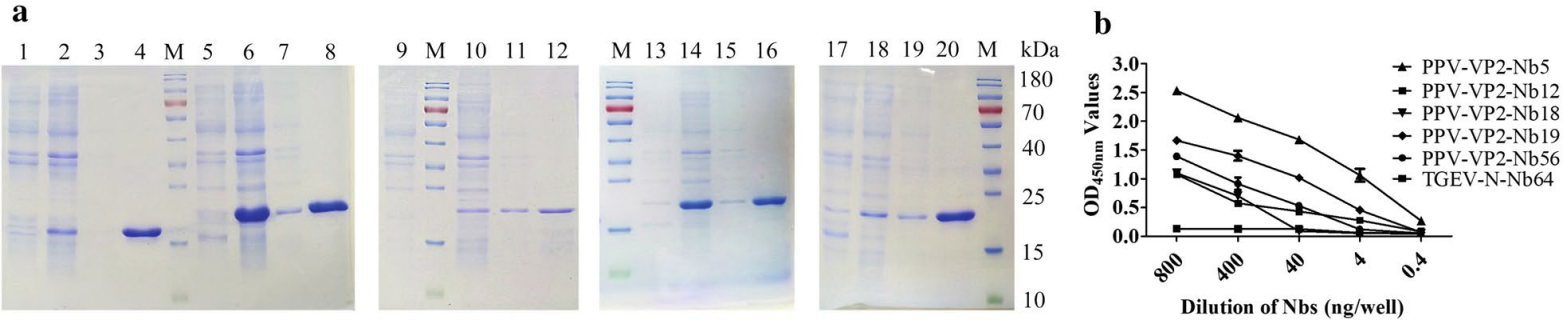
Expression, purification and identification of the 5 recombinant nanobodies against PPV-VP2 protein by prokaryotic system expression. **a** SDS-PAGE analysis of expression and purification of the 5 PPV-VP2 specific nanobodies by *E. coli*. M: Marker; lanes 1, 5, 9, 13, 17: Un-induced of PPV-VP2-Nb5, -Nb12, -Nb18, -Nb19 and -Nb56, respectively; lanes 2, 6, 10, 14, 18: Supernatant of PPV-VP2-Nb5, -Nb12, -Nb18, -Nb19 and -Nb56 after sonication, respectively; lanes 3, 7, 11, 15, 19: Precipitation of PPV-VP2-Nb5, -Nb12, -Nb18, -Nb19 and -Nb56, respectively; lanes 4, 8, 12, 16, 20: Purification of PPV-VP2-Nb5, -Nb12, -Nb18, -Nb19 and -Nb56, respectively. **b** Detection of the 5 recombinant nanobodies specifically binding to the PPV-VP2 protein with the indirect ELISA

Through the sequences alignments by the MegAlign software, the results showed that the five genes encoding the 5 nanobodies were successfully ligated into the pET-25b vector (Additional file 1: Fig. S2). SDS-PAGE analysis showed that PPV-VP2-Nb5, -Nb12, -Nb18, -Nb19, and -Nb56 were successfully expressed with the expected size of 15 kDa and highly purified PPV-VP2-Nb5, -Nb12, -Nb18, -Nb19, and -Nb56 protein were obtained after purification (Fig. 3a). The indirect ELISA results revealed that the 5 expressed nanobodies still specifically bond with PPV-VP2 protein and not with TGEV-N-Nb64 protein, which was expressed with the same vector and system as the negative control (Fig. 3b).

### Production of nanobody-HRP and -EGFP fusions against PPV-VP2 protein

**Fig. 4:**
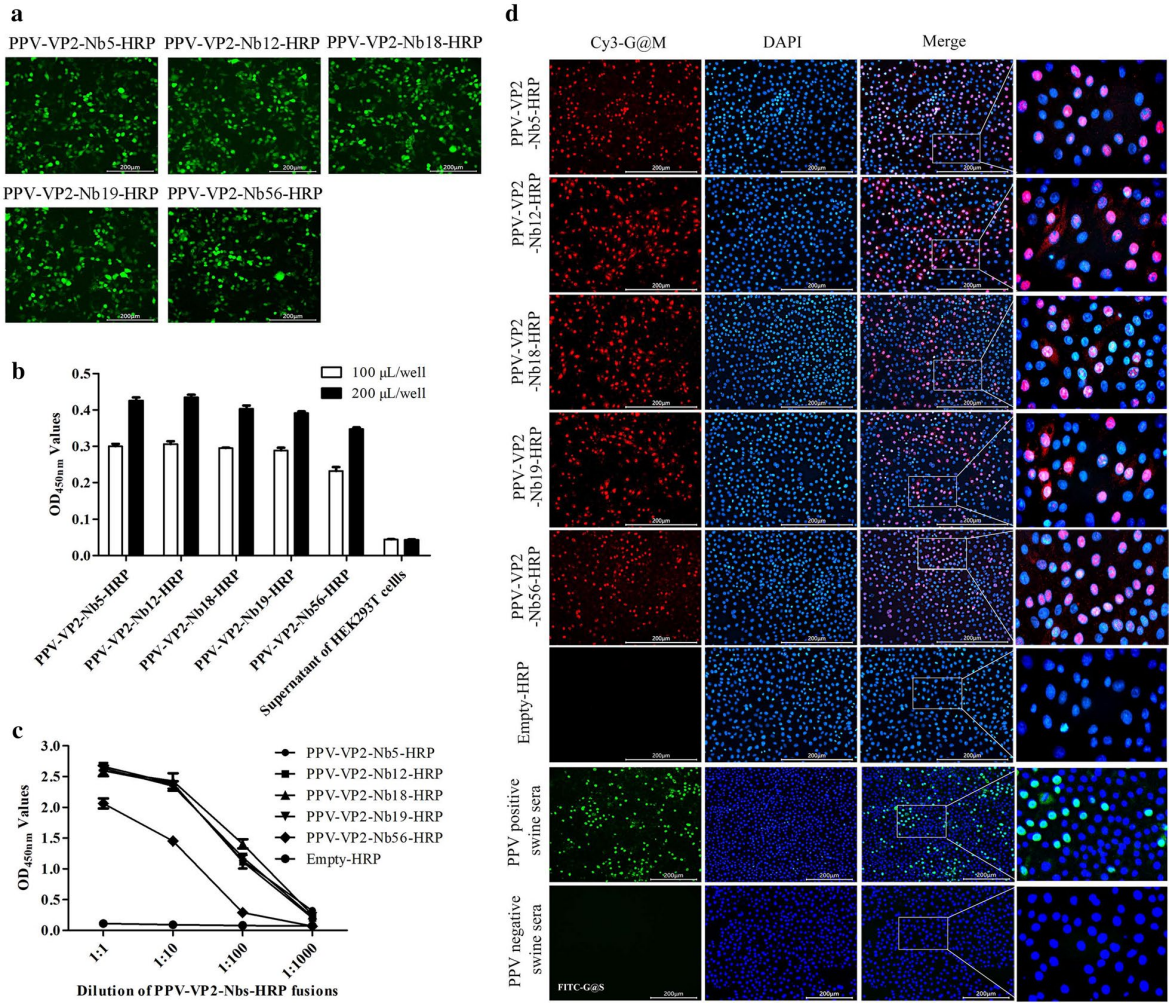
Expression and characterisation of the 5 PPV-VP2-Nbs-HRP fusions in the HEK293T cells. **a** Identification of PPV-VP2-Nbs-HRP expressed in the HEK293T cells by IFA. **b** Detection of the HRP activity in the PPV-VP2-Nbs-HRP fusions secreted into the culture medium of HEK293T cells. **c** Titres of the PPV-VP2-Nbs-HRP in the medium reaction with the PPV-VP2 protein by direct ELISA. The blank vector (Empty-HRP) was as the negative control. **d** Detection of the PPV in the ST cells with PPV-VP2-Nbs-HRP fusions as the first antibody by IFA. PPV positive swine serum as the positive control; PPV negative swine serum and supernatant of HEK293T cells transfected with blank vector as the negative control

The 5 nanobody-HRP fusions against PPV-VP2 protein were successfully expressed in the HEK293T cells (Fig. 4a) and secreted into the medium (Fig. 4b). The direct ELISA results showed that the 5 nanobody-HRP fusions still reacted with the PPV-VP2 protein (Fig. 4c) and were subsequently named PPV-VP2-Nb5-HRP, -Nb12-HRP, -Nb18-HRP, -Nb19-HRP, and -Nb56-HRP. The titres of PPV-VP2-Nb5-HRP, -Nb12-HRP, -Nb18-HRP, and -Nb19-HRP in the medium were 1:100, while the titre of PPV-VP2-Nb56-HRP was 1:10 (Fig. 4c). In addition, the IFA results indicated that all 5 nanobody-HRP fusions could be used to detect PPV in ST cells (Fig. 4d).

**Fig. 5:**
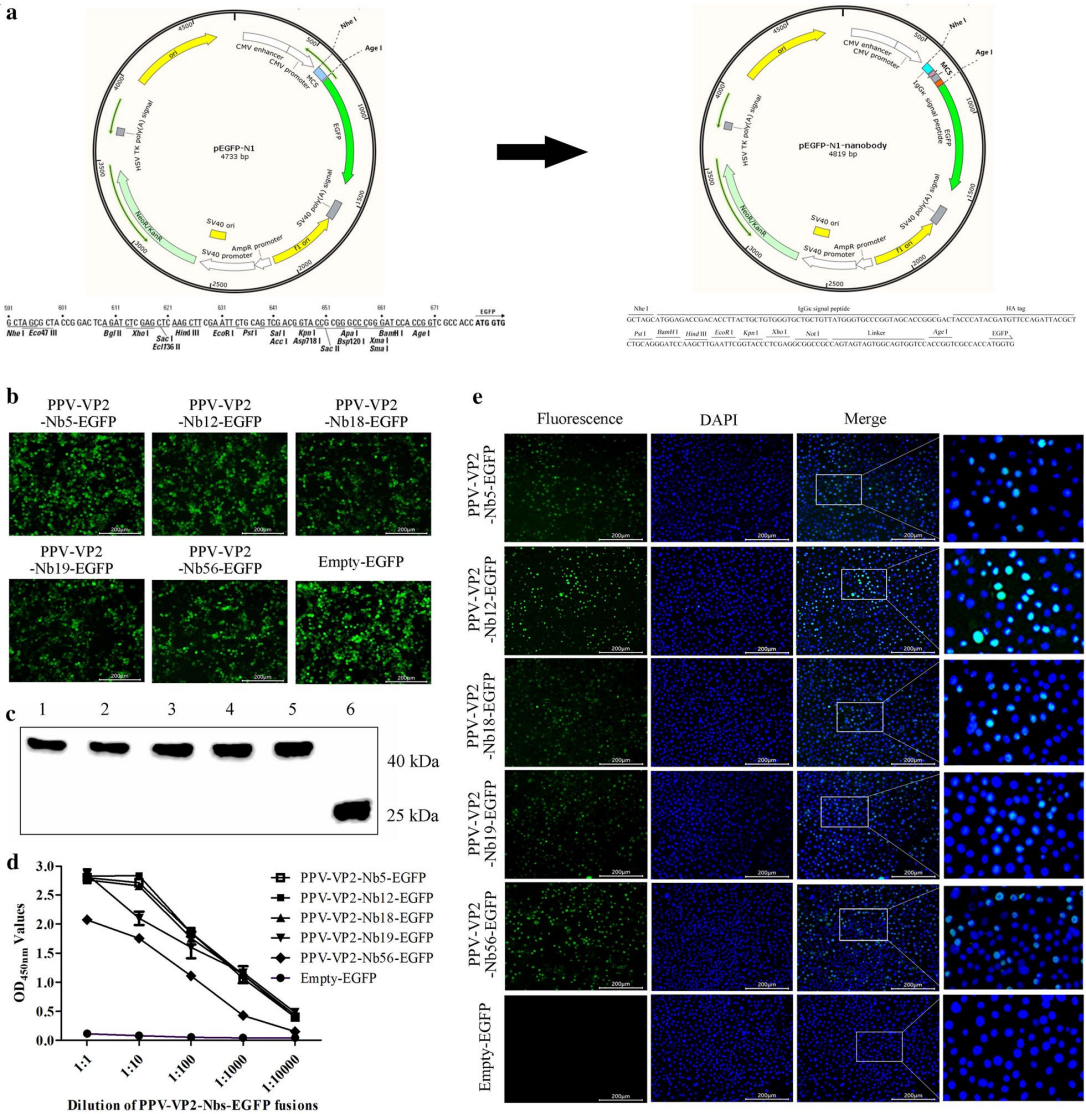
Expression and characterisation of the 5 nanobody-EGFP fusions in the HEK293T cells. **a** Schematic presentation of the commercial vector pEGFP-N1 changed into the vector to insert the main genes encoding IgG signal peptide and multiple cloning site (MCS). **b** Detection of the expression of PPV-VP2-Nbs-EGFP in the HEK293T cells with direct fluorescence assay. **c** Detection of the PPV-VP2-Nbs-EGFP secreted into the culture medium of HEK293T cells by Western blot. Lanes 1-6: PPV-VP2-Nb5-EGFP, PPV-VP2-Nb12-EGFP, PPV-VP2-Nb18-EGFP PPV-VP2-Nb19-EGFP, PPV-VP2-Nb56-EGFP and Empty-EGFP (blank vector as negative control), respectively. **d** Titres of the PPV-VP2-Nbs-EGFP in the medium reacting with the PPV-VP2 protein by indirect ELISA. **e** Detection of PPV in the ST cells with the 5 PPV-VP2-Nbs-EGFP fusions by direct fluorescent assay

To produce the nanobody-EGFP fusions, the expression vector was firstly designed and constructed. A secreting signal sequence (the human IgG kappa chain), HA tag, multiple cloning site (MCS) and short linker sequence were successfully inserted into the pEGFP-N1 vector, which was named pEGFP-N1-nanobody vector (Fig. 5a). Then, the 5 VHH genes were separately inserted into the vector and positive clones were transfected into HEK293T cells, direct fluorescent observations exhibited expression of the 5 nanobody-EGFP fusions (Fig. 5b). Western blot results demonstrated that they were all secreted into the medium (Fig. 5c). In addition, the indirect ELISA also showed that the 5 nanobody-EGFP fusions still reacted with the PPV-VP2 protein and were named PPV-VP2-Nb5-EGFP, -Nb12-EGFP, -Nb18-EGFP, -Nb19-EGFP and -Nb56-EGFP. The titres of PPV-VP2-Nb5-EGFP, -Nb12-EGFP, -Nb18-EGFP, and -Nb19-EGFP were all 1:1000 and the one of PPV-VP2-Nb56-EGFP was 1:100 (Fig. 5d). The results of direct immunofluorescent assay further suggested that the 5 nanobody-EGFP fusions could detect PPV in ST cells (Fig. 5e).

### Nanobody-EGFP fusion as a probe to detect PPV by direct immunofluorescent assay

**Fig. 6:**
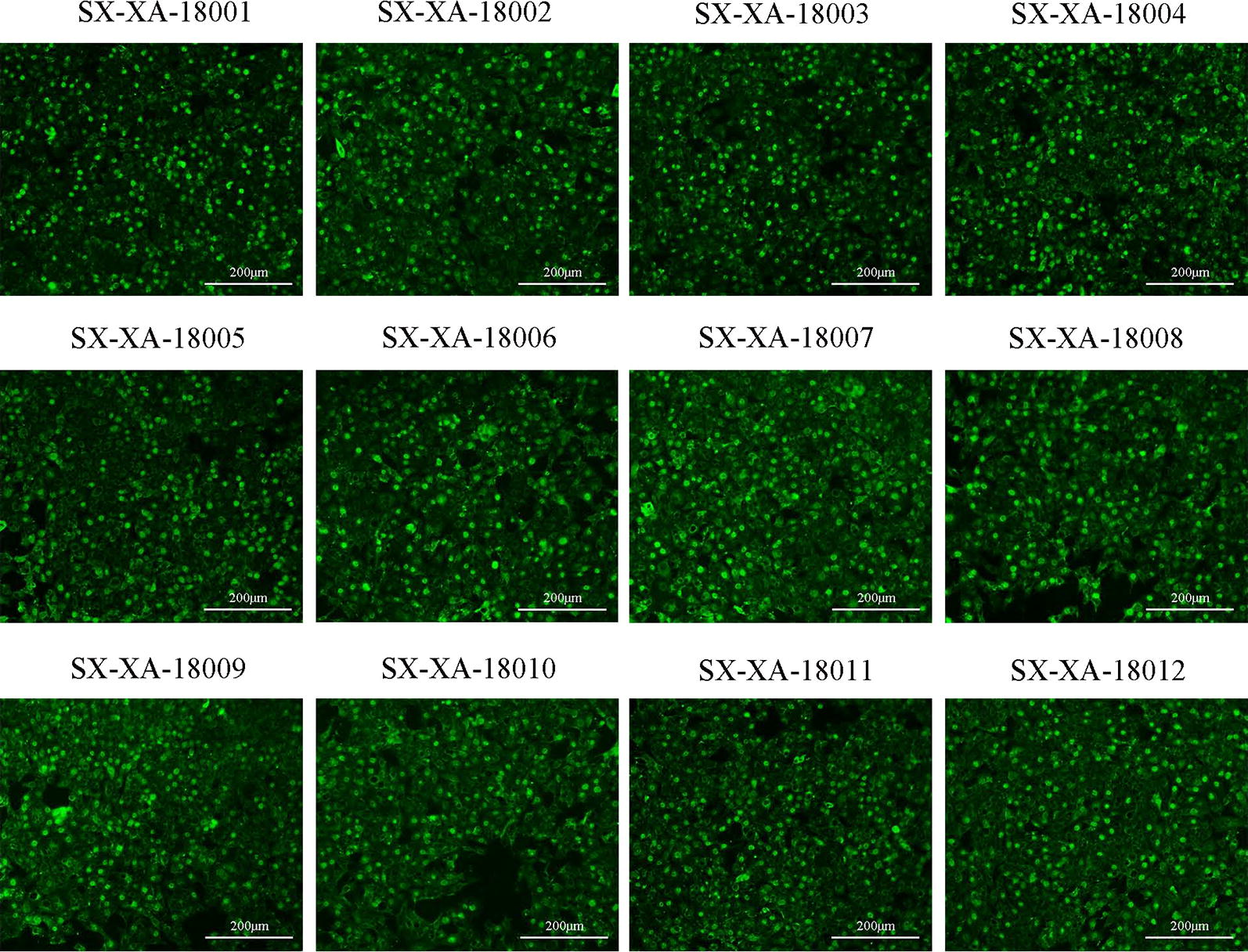
Detection of 12 clinical PPV isolates infecting the ST cells by the direct immunofluorescent assay with the PPV-VP2-Nb12-EGFP fusion as a probe

Among the five nanobody-EGFP fusions, the strongest fluorescence was observed under microscopy when the PPV-VP2-Nb12-EGFP fusion was used (Fig. 5e). Therefore, PPV-VP2-Nb12-EGFP fusion was selected as the probe to detect 12 PPV isolates in ST cells by direct immunofluorescent assay. The results revealed that all 12 PPV isolates were positive (Fig. 6), suggesting that the PPV-VP2-Nb12-EGFP fusion may be universally used to detect PPV in ST cells without using a traditional fluorescent-secondary antibody.

### Nanobody-HRP fusion as a probe for developing sandwich ELISA-like immunoassay to detect PPV

The results showed that the P/N value was highest (7.89) when PPV-VP2-Nb19 and PPV-VP2-Nb56-HRP fusions were selected for pairing in sandwich ELISA-like immunoassay (Table 2). Then, the optimisation parameters were determined, from which the P/N value was highest (20.80) when 4000 ng/well PPV-VP2-Nb19 and a 1:100 dilution of PPV-VP2-Nb56-HRP fusion were used (Table 3).

**Table 2:** Determination of the best pairs of nanobodies, as the capture antibody, and nanobody-HRP fusions as the detection antibody for the sandwich ELISA-like immunoassay to detect PPV by orthogonal assay

Different nanobody-HRP fusions as detection antibody	Samples	Different nanobodies expressed by *E. coli* as capture antibody				
		PPV-VP2-Nb5	PPV-VP2-Nb12	PPV-VP2-Nb18	PPV-VP2-Nb19	PPV-VP2-Nb56
PPV-VP2-Nb5-HRP	Positive	0.83	0.60	0.42	0.52	0.49
	Negative	0.19	0.21	0.20	0.21	0.22
	P/N	4.35	2.87	2.10	2.49	2.20
PPV-VP2-Nb12-HRP	Positive	0.78	0.36	0.28	0.29	0.24
	Negative	0.22	0.19	0.21	0.24	0.19
	P/N	3.49	1.88	1.30	1.21	1.26
PPV-VP2-Nb18-HRP	Positive	0.64	0.30	0.23	0.25	0.35
	Negative	0.23	0.21	0.19	0.20	0.21
	P/N	2.80	1.45	1.24	1.25	1.62
PPV-VP2-Nb19-HRP	Positive	0.67	0.35	0.26	0.38	0.30
	Negative	0.19	0.24	0.23	0.25	0.18
	P/N	3.49	1.48	1.12	1.50	1.67
PPV-VP2-Nb56-HRP	Positive	1.23	0.77	0.51	*1.50*	0.64
	Negative	0.21	0.22	0.21	*0.19*	0.23
	P/N	5.86	3.52	2.45	*7.89*	2.79

Italic is the best conditions

**Table 3:** Optimised amount of PPV-VP2-Nb19 as the capture antibody and dilution of PPV-VP2-Nb56-HRP fusions in the medium as the detection antibody using the developed sandwich ELISA-like assay

Different amounts of PPV-VP2-Nb19 (ng/well)	Samples	Dilutions of the PPV-VP2-Nb56-HRP fusions as detection antibody				
		1:1	1:10	*1:100*	1:1000	1:10,000
8000	Positive	0.88	0.92	0.96	0.61	0.42
	Negative	0.06	0.05	0.05	0.06	0.06
	P/N	14.67	18.40	19.2	10.17	7.00
*4000*	Positive	0.75	0.80	*1.04*	0.50	0.34
	Negative	0.06	0.05	*0.05*	0.06	0.05
	P/N	12.50	16.00	*20.80*	8.33	6.8
2000	Positive	0.73	0.77	0.87	0.46	0.30
	Negative	0.05	0.06	0.05	0.05	0.04
	P/N	14.6	12.83	17.4	9.20	7.5
1000	Positive	0.74	0.72	0.82	0.43	0.25
	Negative	0.05	0.04	0.05	0.06	0.06
	P/N	14.8	18.00	16.40	7.17	4.17

Italic represents the best conditions

After the 200 negative samples were detected with the developed assay, the mean value of OD_450nm_ values and SD were determined to be 0.0576 and 0.0126, respectively. Further, the cut-off value of the assay was 0.0954, which means the value was above 0.0954 as positive and vice versa for a negative test.

**Fig. 7:**
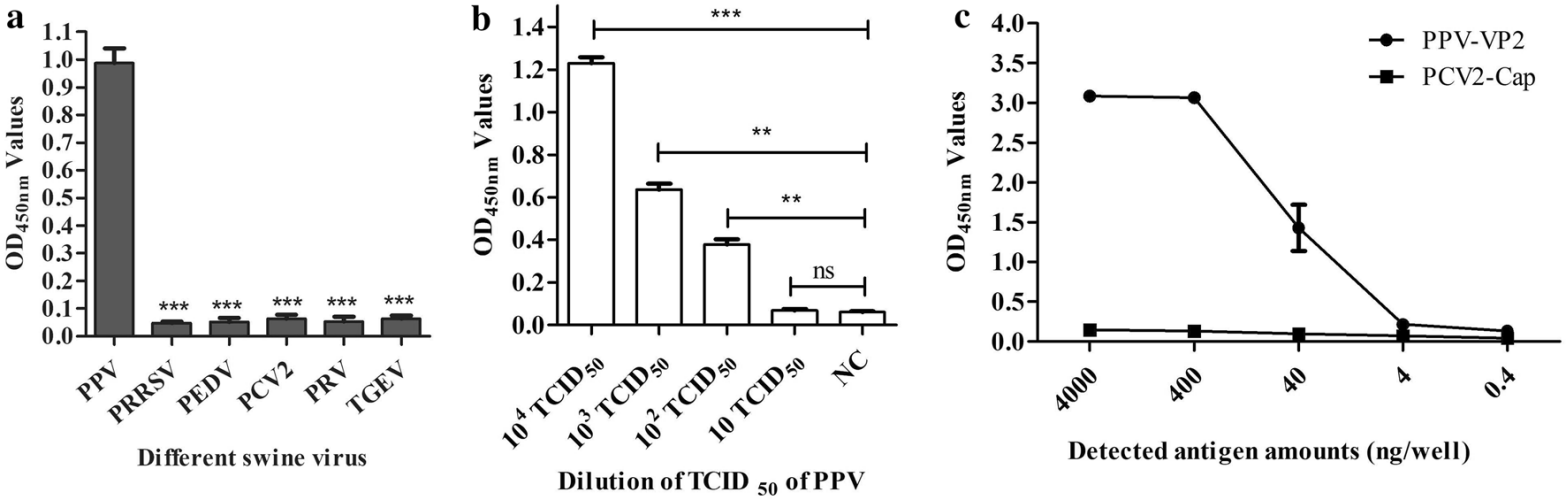
Specificity and the minimum limitation of the developed sandwich ELISA-like immunoassay using nanobody as the capture antibody and nanobody-HRP fusion as the detection antibody for detecting PPV and PPV-VP2 protein. **a** Specificity analysis of the developed sandwich ELISA-like assay. **b** Low limitation of the developed sandwich ELISA-like assay. **c** The minimum amount of PPV-VP2 protein detected by the developed sandwich ELISA-like assay. The PCV2-Cap protein expressed with the same system was as the negative control

Using the assay to detect other pig disease viruses (PRRSV, PEDV, PCV2, PRV, and TGEV), the results showed that the OD_450nm_ values of these viruses were all below 0.0954 (Fig. 7a).

For the low detection limit of the assay, the results showed that the minimum amount of PPV was 100 TCID_50_ per 100 μL (Fig. 7b), which was same as the amount of 5.41 × 10^6^ copies/μL determined by real-time PCR and the amount of the recombinant PPV-VP2 protein expressed by *E. coli* was 40 ng (Fig. 7c).

From the 64 clinical samples, 23 samples (16 faeces and 7 sera) tested positive for PPV using the assay, indicating that the assay could be used to detect PPV in clinical samples.

### Agreements among nanobody-HRP and -EGFP fusions as the probes in the immunoassay, real-time PCR and commercial monoclonal-based sandwich ELISA

For the clinical samples, the agreements of the direct immunofluorescent assay with real-time PCR and with the commercial monoclonal antibody-based sandwich ELISA were 81.5% and 90.8%, respectively (Table 4). Moreover, based on the above detection results, the agreements of the developed sandwich ELISA-like assay with the real-time PCR and with the commercial monoclonal antibody-based sandwich ELISA were 92.1% and 81.5%, respectively (Table 4).

**Table 4:** Separate comparisons of the developed sandwich ELISA-like assay using nanobody-HRP fusion as the detection antibody and direct immunofluorescent assay using nanobody-EGFP fusion with real-time PCR and with a commercial monoclonal antibody-based sandwich ELISA by detecting PPV from the clinical samples

Assay		Number	Real-time PCR		Agreement (%)	Kappa value	Commercial monoclonal antibody-based sandwich ELISA		Agreement (%)	Kappa value
			+	−			+	−		
Developed sandwich ELISA-like assay	+	23	23	0	92.1	0.838	14	9	81.5	0.6
	−	41	5	36			2	39		
Direct immunofluorescent assay	+	17	17	0	81.5	0.635	14	3	90.8	0.796
	−	47	11	36			2	45		

“+” represents positive; “−” as negative

For the samples from the challenged pigs with PPV, 4 fecal samples at 10 and 14 dpi were positive and 5 sera at 7 and 10 dpi were positive with the developed sandwich ELISA-like assay (Table 5). For the direct immunofluorescent assay, 2 fecal samples at 10 dpi and 3 sera at 7 and 10 dpi were positive (Table 5). For real-time PCR, 8 fecal samples at 5, 7, 10, and 14 dpi and 7 sera at 5, 7, and 10 dpi were positive (Table 5). For the commercial monoclonal antibody-based sandwich ELISA, 2 fecal samples at 10 dpi and 1 serum at 10 dpi were positive (Table 5).

**Table 5:** Comparisons between the developed sandwich ELISA-like assay, direct immunofluorescent assay, real-time PCR and commercial monoclonal antibody-based sandwich ELISA by detecting the sequential fecal and serum samples from pigs infected with PPV

Different assays for detecting PPV	Samples from the different dpi of 3 challenged pigs (Number positive of faeces**/**number positive of sera)						
	3	5	7	10	14	21	28
Developed sandwich ELISA-like assay	0/0	0/0	0/2	3/3	1/0	0/0	0/0
Direct immunofluorescent assay	0/0	0/0	0/1	2/2	0/0	0/0	0/0
Real-time PCR	0/0	2/2	3/2	2/3	1/0	0/0	0/0
Commercial monoclonal antibody-based sandwich ELISA	0/0	0/0	0/0	2/1	0/0	0/0	0/0

## Discussion

Antibodies are considered one of the most effective biomolecules to be used for detection methodologies and applications [42–44]. Due to their particular characteristics, such as high affinity and specificity, a large variety of immunoassay formats implementing antibodies as reagents have been developed for disease diagnoses. However, there is an increasing demand to improve some properties of conventional antibodies that are currently being used. Nanobodies have arisen as a substitute to conventional antibodies and show great potential when used as tools in the diagnostic field [45–48]. One of the main advantages of nanobodies is that several tags can be fused in their tertiary structure by recombinant technology [9–11, 13]. Based on this advantage, PPV-VP2 specific nanobody-HRP and -EGFP fusions were used for the first time as probes to develop immunoassays for PPV detection in this study. The two assays demonstrated higher sensitivity, more specificity and simple operation compared with the traditional antibodies-based immunoassays.

Another advantage of nanobodies is their large-scale production with good yields. The screening of nanobodies and construction of recombinant plasmids may be time consuming for the fresh hand. When the above procedures were finished, the following operations are simple for large scale production. In this work, PPV-VP2 specific nanobody-HRP and -EGFP were secreted into the cell medium for direct detection after the positive plasmids were transfected into HEK293T cells. The procedure eliminates the use of commercial secondary antibodies and shortens detection time in the immunoassays. Importantly, HEK293T cell lines stably expressing the PPV-VP2 specific nanobody-HRP and -EGFP fusions can be constructed by cell domestication technology, decreasing the cost and simplifying production of the two fusions. Overall, we believe that the two developed nanobody-HRP and -EGFP-based immunoassays for detecting PPV have great developmental prospects for further commercial production.

With the development of swine industry, the infection rate of PPV has shown a clear upward trend, and the reproductive failure in sows caused by infection has increased worldwide [49]. This is lead to the development of many assays for detecting PPV in samples. Compared with the other reported methods for detecting PPV particles, the developed sandwich ELISA-like immunoassay showed higher sensitive (100 TCID_50_ per 100 µL). However, compared with the reported PCR or real-time PCR for detecting the genes of PPV, the developed assay showed lower sensitive, which the limitation was only 5.41 × 10^6^ copies/μL because the genes of the PPV were not amplified [31, 39, 50, 51]. Although the PCR or real-time PCR is widely used due to its high specificity, sensitivity, and accuracy, they requires a complicated operation and is susceptible to contamination by aerosols, resulting in false positives [33]. In this study, the first time developed sandwich ELISA-like immunoassay, employing the nanobody as the capture antibody and nanobody-HRP fusion as the detection antibody, showed high agreement with real-time PCR and could be used to detect PPV from the clinical samples. Comparatively, the developed assay shortens detection time, simplifies the operation, and eliminates the need for HRP-labelled secondary antibodies that are required in the commercial monoclonal antibody-based sandwich ELISA. These advantages further suggest that the developed sandwich ELISA-like assay can be universally used to surveil PPV infection in pig flock.

Virus isolation following detection with IFA is considered as “gold” standard for detecting PPV in the samples [49]. However, for the conventional antibodies-based IFA, a fluorescent labelled secondary antibody must be used, which is both time-consuming and costly. In this study, for the first time, a PPV-VP2 specific nanobody-EGFP fusion was developed and used to detect PPV in ST cells, eliminating the use of commercial secondary antibodies. In addition, the direct fluorescent assay, implementing the PPV-VP2 specific nanobody-EGFP fusion as a probe, can also be used to detect the different PPV isolates in ST cells. This further demonstrates that the fusion can be universally applied to detect PPV in laboratory and clinical testing.

## Conclusion

Nanobody-reporter fusions can circumvent many limitations of conventional antibodies for diagnostic application. In the present study, five PPV-specific nanobodies were firstly produced from an immunised Bactrian camel. And then, PPV-VP2 specific nanobody-HRP and -EGFP fusions were the first time to design and produce by transfection of HEK293T cells. Subsequently, a sandwich ELISA-like immunoassay for detecting PPV in the samples was firstly developed using the nanobody as a capture antibody and nanobody-HRP fusion as the detection antibody. The developed assay showed good agreement with real-time PCR for detecting PPV in the samples. In addition, a direct fluorescent assay using nanobody-EGFP as a probe was also firstly developed to detect PPV in ST cells. Compared with conventional antibodies-based immunoassays to detect PPV from clinical samples, the two assays eliminate the use of commercial secondary antibodies and shorten detection time. Conclusively, this work provides a novel technique for developing and using a nanobody-based direct sandwich ELISA and direct fluorescent assay to detect, subsequently, prevent further PPV infection in pig flock.

## Supplementary information

**Additional file 1: Fig. S1**Sequence alignment between the VP2 genes in the positive plasmid with the target one. **Fig. S2.** Sequence analysis of the different genes encoding the nanobodies in the recombinant vectors pET-25b-VP2-Nbs with the target ones from the screening VHH genes.

## Data Availability

All data generated or analyzed during this study are included in the article.
