# Supplementary material for: Nanobody‑horseradish peroxidase and -EGFP fusions as reagents to detect porcine parvovirus in the immunoassays
Source: J Nanobiotechnology. 2020 Jan 7;18:7. doi: 10.1186/s12951-019-0568-x (PMC6945459; doi:10.1186/s12951-019-0568-x)
Supplement: Supplementary file 1 — Additional file 1: Fig. S1Sequence alignment between the VP2 genes in the positive plasmid with the target one. Fig. S2. Sequence analysis of the different genes encoding the nanobodies in the recombinant vectors pET-25b-VP2-Nbs with the target ones from the screening VHH genes. [file 12951_2019_568_MOESM1_ESM.docx]

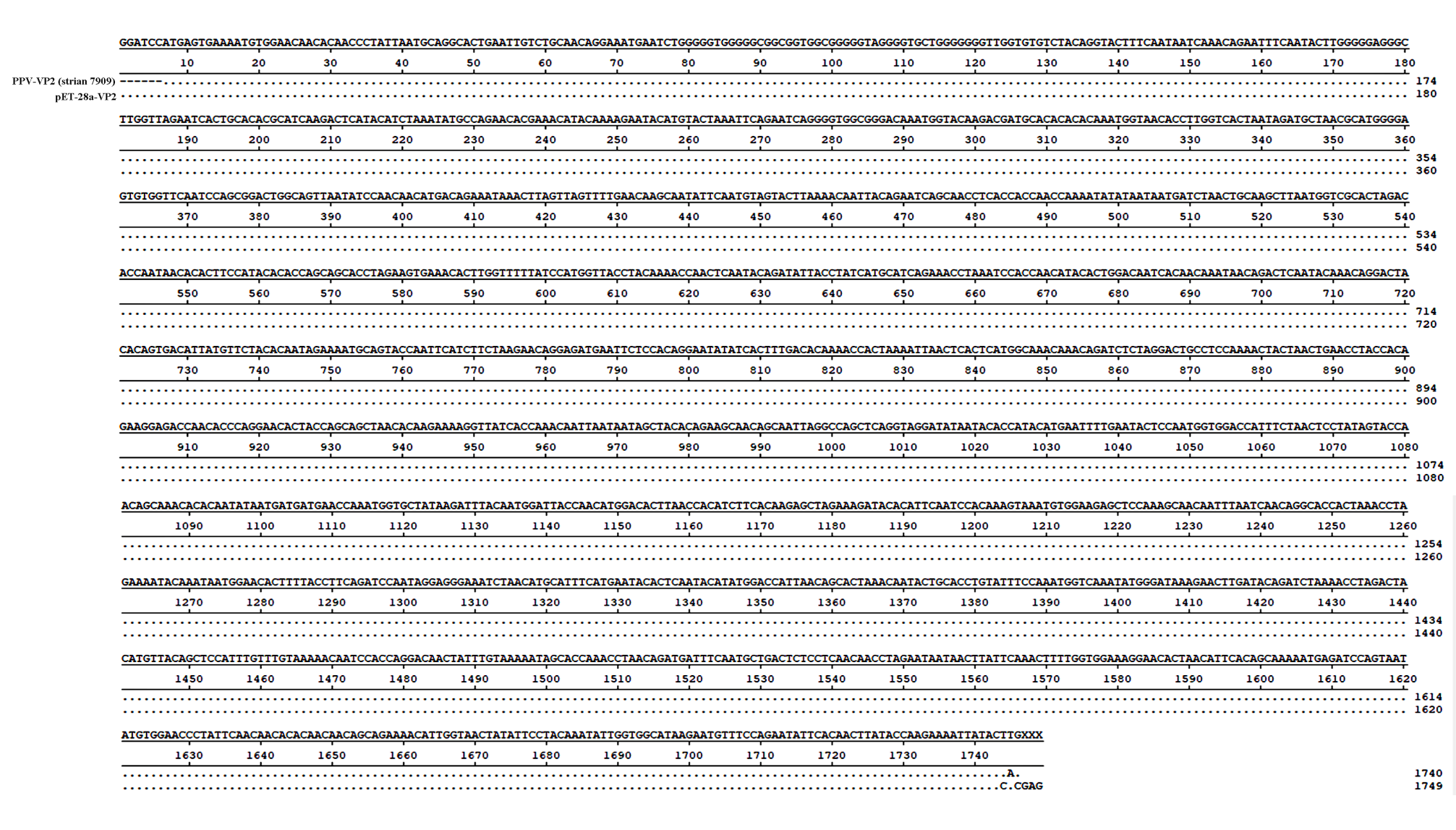


**Fig. S1** Sequence alignment between the VP2 genes in the positive plasmid and the target one.


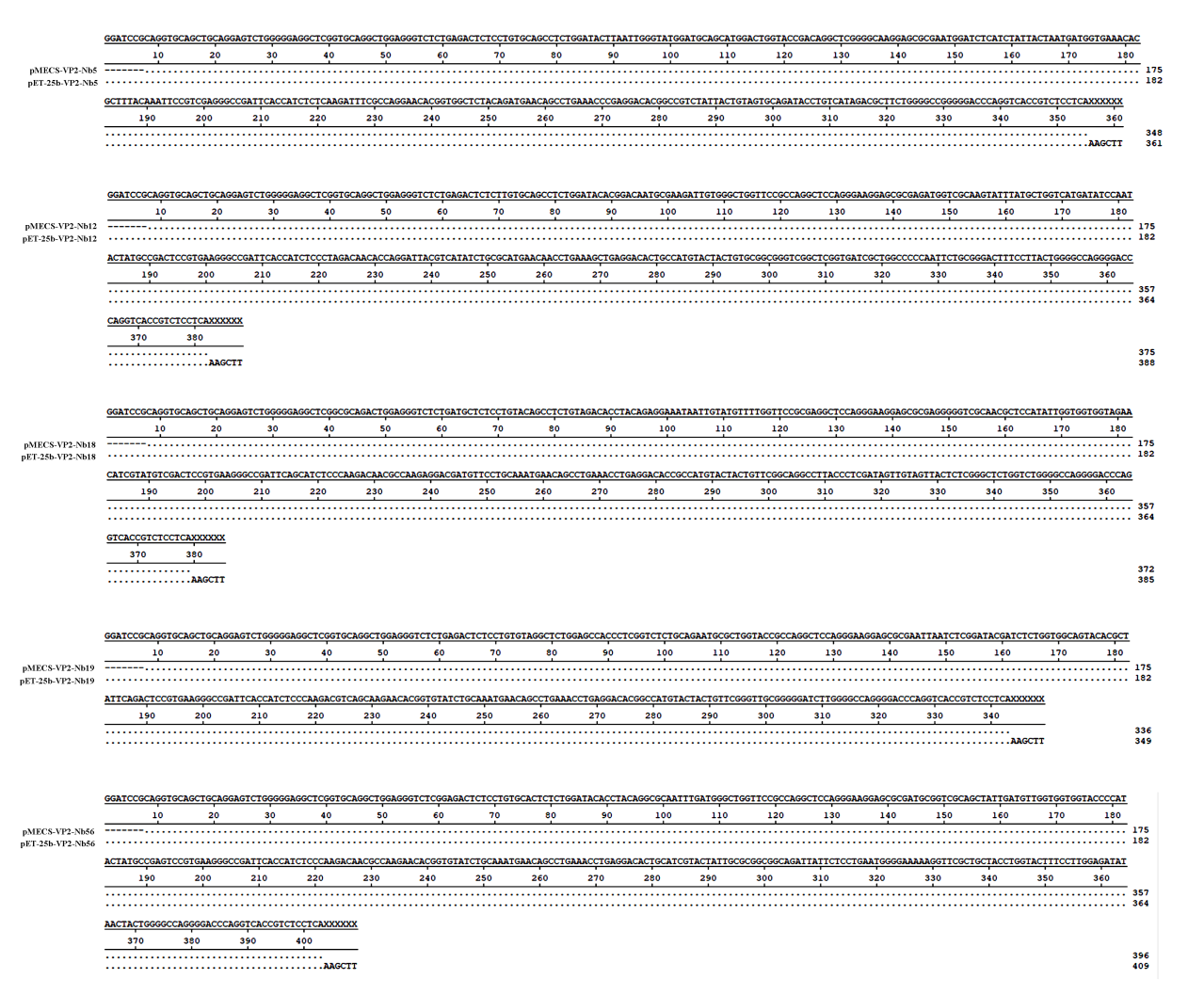


**Fig. S2** Sequence analysis of the different genes encoding the nanobodies in the recombinant pET-25b-VP2-Nbs with the screening VHH genes.
